# Long-term monitoring reveals climate-driven seasonal dynamics of *Zeugodacus cucurbitae* (Coquillett) in Tanzania and prospects for pest control

**DOI:** 10.1186/s12862-026-02536-6

**Published:** 2026-06-01

**Authors:** Maulid W. Mwatawala, Marc De Meyer, Jackline Bakengesa, Mwajuma K. Zinga, Joseph O. Ruboha

**Affiliations:** 1 Department of Crop Science and Horticulture, Sokoine University of Agriculture, Morogoro, Tanzania; 2 Biology Department, Royal Museum for Central Africa, Tervuren, Belgium; 3 Biology Department, The University of Dodoma, Dodoma, Tanzania; 4 Department of Agricultural Sciences, Sokoine University of Agriculture, Mizengo Pinda Campus, Katavi, Tanzania

**Keywords:** *Zeugodacus cucurbitae*, Long-term monitoring, Seasonality, Altitude, Tanzania

## Abstract

*Zeugodacus cucurbitae* (Coquillett) is a major and economically important pest of cucurbit crops, causing substantial losses to farmers across the tropics and subtropics. We analyzed a long-term trapping dataset (October 2004 - February 2012), collected from various agro-ecological settings in the Morogoro Region of Tanzania. This included a warm lowland orchard at Sokoine University of Agriculture (SUA) (526 m.a.s.l.) and a cooler highland orchard at Nyandira (1580 m.a.s.l.), as well as five additional locations along the same altitudinal gradient. Adult male *Z. cucurbitae* were monitored using cue-lure–baited modified McPhail traps. The abundance data was standardised as Flies per Trap per Day (FTD). Climatic-driven data were compiled from multiple sources. Results revealed significant spatial variation in *Z. cucurbitae* abundance along the altitudinal gradient (*p* < 0.0001), with sustained high densities at SUA, Kibundi and Mlali, intermediate levels at Langali, and chronically low abundance at Nyandira and Visada. Inter-annual variation was significant (*p* < 0.0001), with marked regional amplification in 2011–2012, whereas month-of-year effects were not significant (*p* = 0.7925). Directional increases occurred at Kibundi and Langali, while SUA and Mlali showed episodic dynamics and Nyandira remained stable at low levels. Abundance was negatively associated with elevation and positively associated with temperature (both *p* < 0.0001). Management should therefore continue in low-altitude areas, while regular monitoring, especially in high-altitude areas, is necessary to detect any steady increase in *Z. cucurbitae* abundance.

**Supplementary Information:**

The online version contains supplementary material available at 10.1186/s12862-026-02536-6.

## Introduction

The melon fruit fly *Zeugodacus cucurbitae* (Coquillett) (Diptera: Tephritidae) is a major and economically important invasive pest species of cucurbit crops that cause serious losses to farmers, across the tropics and sub-tropics [1]. *Z. cucurbitae* originated from the Indian sub-continent and was introduced to different parts of the world [2], and became widely distributed in Africa, Indian Ocean islands, Australasia, and the Pacific Islands, including Hawaii, where it is an impediment to cucurbit production [3, 4]. In Africa, the first record of *Z. cucurbitae* dates back to 1936 in Tanzania [4, 5], and current records show that the species is now present across most tropical and subtropical regions of the continent [6].

Damage caused by *Z. cucurbitae* is closely linked to its reproductive biology and feeding behaviour, as female flies oviposit into young or developing fruits, where the emerged larvae feed on tissues leading to premature fruit drop, secondary microbial infection, and rapid fruit rotting that render the product unmarketable [1, 3]. Owing to its high fecundity, short generation time [7–9], and strong association with cucurbit crops [4], *Z. cucurbitae* is major constraint to vegetable production in tropical and subtropical regions due rapid population build-up and persistence across seasons.

The biology and development of *Z. cucurbitae* are strongly influenced by environmental conditions. Experimental studies demonstrate that developmental rate, survival, and reproductive output vary with temperature and host species [9–11], with faster development and higher survivorship occurring at favorable warmer temperatures that are within optimal range of the species. These temperature-dependent traits help explain the pronounced seasonal and geographical variation observed in field populations. Temperature directly influences development and fecundity, while rainfall and relative humidity affect adult activity, larval survival, and fruit availability [12, 13]. Seasonal variation in natural enemy activity may further modulate population growth [14]. De Meyer et al. [4] reported that regional variation in the abundance, seasonality, and interactions of *Z. cucurbitae* with indigenous *Dacus* species that indicates strong geographical variability in population phenology.

Contrasting patterns have been reported for *Z. cucurbitae* population dynamics in different regions of Africa suggesting the complexity of its ecological responses. In West Africa, Gnanvossou et al. [13] reported that populations increased with rising temperatures, particularly during the hotter months of the year, and declined at lower temperatures during the colder months. In contrast, in Morogoro East Africa, Mwatawala et al. [12] reported that peak populations occurred during the dry period (July – September), when temperature and relative humidity were also low.

Competition between the invasive *Z. cucurbitae* and indigenous cucurbit-infesting *Dacus* species further contribute to these contrasting patterns, influencing seasonal abundance and species dominance in cucurbit agroecosystems. In these systems, *Z. cucurbitae* commonly co-occurs with native species such as *Dacus ciliatus* Loew, *Dacus vertebratus* (Bezzi), and *Dacus bivittatus* (Bigot). For example, Gnanvossou et al. [13] reported dominance of *Z. cucurbitae* over native *Dacus* species based on parapheromone trapping, a method that may underestimate species that are weakly attracted or not attracted to these lures. From host fruit infestation studies, contrasting dominance patterns were also evident, with *Z. cucurbitae* dominating in East Africa [15–17], whereas indigenous *Dacus* species remain dominant or co-dominant in parts of West Africa [18, 19].

Understanding these population dynamics is critical for designing effective, climate-responsive integrated pest management (IPM) strategies. Effective IPM depends on aligning interventions, such as sanitation, baiting, biological control, and attract-and-kill strategies, with seasonal peaks in adult activity and fruit infestation [15, 20–24]. However, long-term empirical datasets that captures spatial, seasonal, and interannual population dynamics of *Z. cucurbitae* in African agroecosystems, remain limited.

Against this background, the present study aimed to assess the spatio-temporal population dynamics of *Z. cucurbitae* and examine their relationships with key meteorological variables. We hypothesized that populations would exhibit pronounced seasonal and interannual fluctuations driven primarily by temperature and rainfall, and modified by seasonal fruit availability and interspecific competition, although the latter were not quantified by this study. We, further, hypothesized that higher abundance occurs during warm and humid periods.

## Methodology

### Study sites and their elevation

**Fig. 1:**
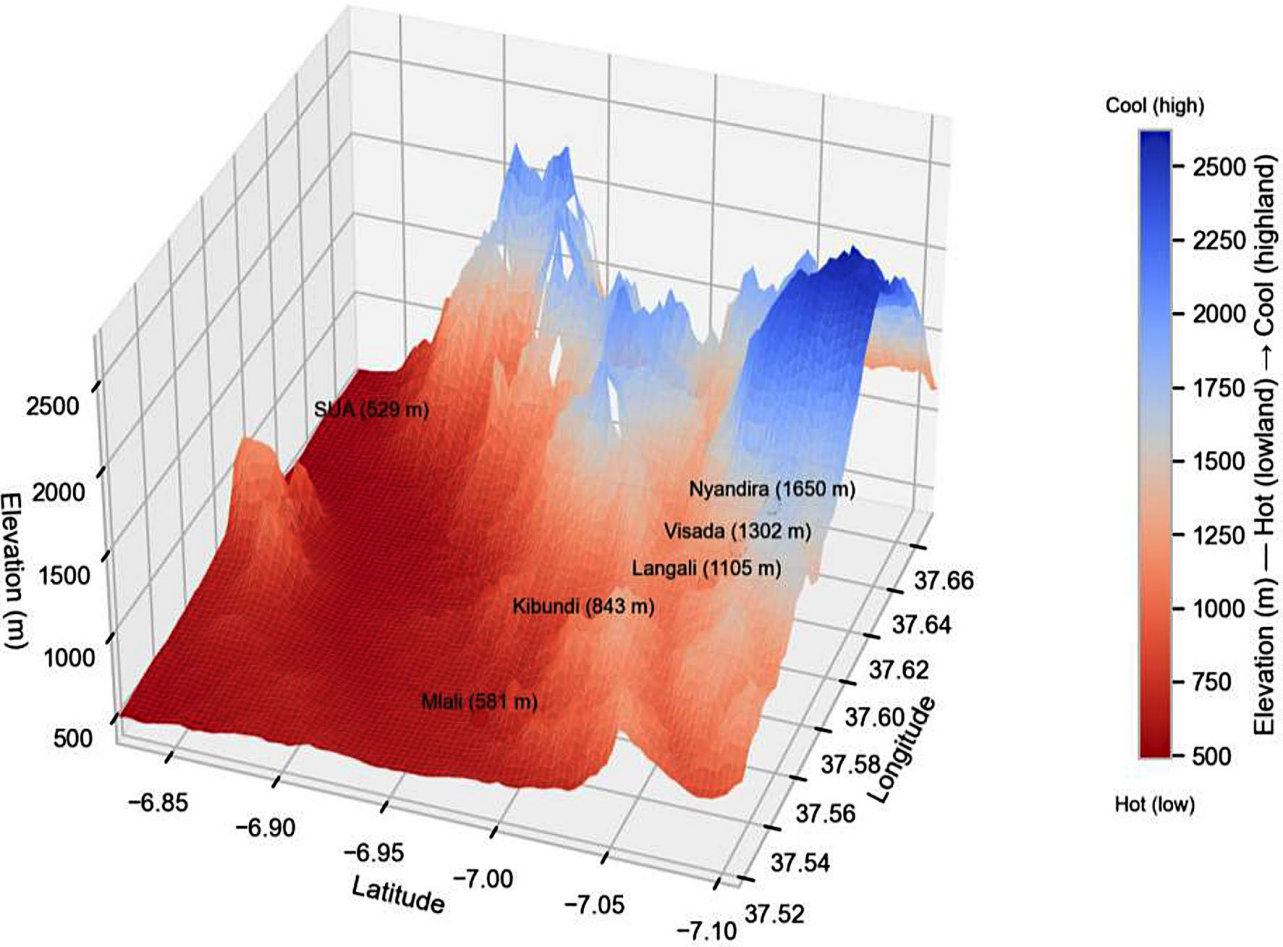
3D elevation surface around the six monitoring sites (SRTM DEM), highlighting the lowland–highland gradient

Monitoring of *Z. cucurbitae* was carried out in the Morogoro Region of Tanzania along an altitudinal gradient, from a lowland of 526 m above sea level (m.a.s.l.), to a highland of 1580 m.a.s.l. The study area encompassed six monitoring sites, with each site representing distinct agro-ecological conditions (Table 1). The additional sites; Mlali, Kibundi, Langali, and Visada were located along the same altitudinal gradient; between the initial lowland horticulture orchard at Sokoine University of Agriculture (SUA) (526 m.a.s.l.) and highland conditions at Nyandira (1580 m.a.s.l), in order to broaden the spatial coverage. (Fig. 1).

**Table 1:** The study sites along altitudinal gradient in the Morogoro Region, Tanzania

Locality	Latitude	Longitude	Elevation (m.a.s.l.)	Monitoring period	No. of traps
SUA (Sokoine University of Agriculture)	6°51′S	37°39′E	526	Oct 2004–Feb 2012	5
Mlali	7°00′S	37°35′E	560	Oct 2008–Feb 2012	3
Kibundi	7°01′S	37°34′E	670	Oct 2008–Feb 2012	3
Langali	7°03′S	37°34′E	950	Oct 2008–Feb 2012	3
Visada	7°04′S	37°34′E	1250	Oct 2008–Feb 2012	3
Nyandira	7°05′S	37°35′E	1580	Oct 2004–Feb 2012	2

Traps were set up in fruit tree orchards and homestead gardens rather than in cucurbit fields. Since *Z. cucurbitae* is primarily a cucurbit pest, the trapping sites did not represent the primary host environment. Instead, trap placement followed local tree availability to ensure adequate canopy cover and trap height: mango trees were commonly used at lower elevations, and peach, plum, or apple trees at higher elevations. This approach captured adults dispersing through the wider landscape rather than flies directly associated with cucurbit host crops. The relationship between trap catches and actual population levels in nearby cucurbit fields therefore remains indirect, and we acknowledge this limitation in the interpretation of our results.

### Orchard vegetation and host diversity

Host plant composition showed a clear pattern along the altitudinal gradient. At the low-elevation site, the SUA orchard, was dominated by tropical fruit species (see Supplementary material S1 Table 1),

In contrast, the high-elevation Nyandira orchard was dominated by temperate and cool-adapted fruits (Supplementary material S1 Table 2).

Sites along the intermediate transect from Mlali to Visada comprised a mixed smallholder cultivated fields, small orchards, homestead gardens, and fallow land. Across the transect, both cultivated and wild representatives of Cucurbitaceae and Solanaceae were present. Cultivated cucurbits included cucumber (*Cucumis sativus* L.), melon (*Cucumis melo* L.), watermelon (*Citrullus lanatus* (Thunb.) Matsum. & Nakai), pumpkin (*Cucurbita moschata* (Duchesne) Duchesne ex Poir.), luffa (*Luffa acutangula* (L.) Roxb.), and bitter gourd (*Momordica charantia* L.), alongside wild hosts such as wild bitter gourd (*Momordica* cf. *trifoliata* (L.)), bitter cucumber (*M.* cf. *foetida* Schumach.), hedgehog cucumber (*Cucumis dipsaceus* Ehrenb. ex Spach), and related species.

### Trapping and monitoring *Z. cucurbitae*

Adult male *Z. cucurbitae* were monitored using cue-lure–baited modified McPhail traps (Scentry Cie, Billings, MT, USA), a lure that can also attract some *Dacus* species [1], suspended at approximately 2 m height within fruit trees in the orchards. Each trap contained dichlorvos (DDVP; Scentry Cie) as a killing agent. Although *Z. cucurbitae* is primarily a cucurbit-infesting species, traps were placed in orchard trees to provide suitable canopy shade and height for effective para-pheromone trapping, following standard practice for fruit fly surveillance in mixed agricultural landscapes [4, 25].

A preparatory sampling round was conducted at SUA and Nyandira in October 2004 to evaluate field protocols and trap performance as reported by Mwatawala et al. [12] Regular systematic monitoring at both SUA and Nyandira began in October 2005 (Table 1). At SUA, three traps were initially installed in mango (*Mangifera indica* L.), citrus (*Citrus* spp.), guava (*Psidium guajava* L.) trees, and at Nyandira, a single trap was placed in an apple (*Malus domestica* Borkh.),. Additional traps at both sites were introduced in January 2006: at SUA, two traps were added in mango and citrus trees, increasing the total to five; at Nyandira, a second trap was added in a plum (*Prunus domestica* L.) tree. Trapping at additional sites along the altitudinal transect (Mlali, Kibundi, Langali, and Visada) began in October 2008, with three replicate traps deployed at each locality (Table 1).

Sampling continuity differed among sites due to logistical constraints and varied in temporal coverage between the two long-term sites (SUA and Nyandira: October 2005–February 2012) and the four transect sites (Mlali, Kibundi, Langali, Visada: October 2008–February 2012). At SUA, monitoring was conducted continuously except for an interruption between November 2007 and March 2008. At Nyandira, several shorter gaps occurred, primarily between late 2006 and late 2008. Among the transect sites, short interruptions of one to two months occurred at Kibundi, Langali, and Visada due to occasional logistical delays; these gaps were treated as unsampled months rather than as zero-abundance observations. Monitoring across all sites continued until February 2012. Monitoring at all these sites continued through February 2012.

Traps were serviced at regular intervals. Each trap was inspected for captured *Z. cucurbitae* specimens. The specimens were then moved into vials with 70% ethanol for preservation. All captured specimens were transported to the laboratory at SUA for identification and data recording.

The inspections were conducted weekly at SUA. while at Nyandira and the additional transect sites, sampling followed a rotational schedule in which traps were deployed for one week every four weeks. After each sampling round, traps were removed and later reinstalled during the next cycle, among the same trees. Lure plugs and DDVP strips were replaced every four weeks.

### Identification and taxonomic validation

Specimens were first separated from non-target insects and then taxonomically identified to species level using morphological diagnostic characters, under a stereomicroscope, following the taxonomic keys and descriptions of White and Elson-Harris [1]. Identifications were further confirmed by taxonomic expertise available within the research team, including reference to bespoke identification sheets developed for the East African fruit fly fauna during the project [4, 25]. The number of *Z. cucurbitae* individuals per trap was recorded together with the number of days since the previous inspection. This enabled standardization of abundance as Flies per Trap per Day (FTD).

### Climate data and gap management

Climatic data were compiled from several sources to accommodate differing data availability at the two sites and to fill gaps during periods with missing observations. At SUA, monthly rainfall (mm), mean air temperature (°C), and mean relative humidity (%) came from the Tanzania Meteorological Authority (TMA) station for the Morogoro area and were taken as representative of orchard conditions. On-site temperature and relative humidity were also logged using iButton data loggers (Maxim Integrated, Sunnyvale, CA, USA).

At all other locations, air temperature and relative humidity were recorded in the orchard with iButton data loggers [25] and converted to monthly means. Because reliable long-term rainfall measurements were not available at these locations, monthly precipitation values were taken from gridded climate datasets corresponding to each site’s location. When trapping or weather records were incomplete, supplementary climate variables were sourced from reanalysis and gridded products. Monthly temperature and precipitation were extracted from the ERA5 reanalysis via Google Earth Engine, and long-term climatic context was described using WorldClim v2 bioclimatic variables (bio01–bio19). These products were used only for prediction and gap-filling and did not replace TMA or on-site logger observations where those existed. Monthly site-level time series of temperature, rainfall, and relative humidity are shown in Supplementary Figures S1–S3, while the corresponding monthly climatologies are shown in Supplementary Figures S4–S6.

### Abundance index and temporal aggregation

The abundance of fruit flies was standardized as flies per trap per day (FTD) to account for variation in trapping effort. For each inspection interval i, FTD was calculated by dividing the total *Z. cucurbitae* count (C_i_) by the product of the number of traps in use (T_i_) and the number of days since the previous inspection (D_i_):

$$\mathrm{FTD}_\mathrm{i}=\mathrm{C}_\mathrm{i}/\mathrm{(T}_\mathrm{i}\times \mathrm{D}_\mathrm{i)}.$$

Monthly mean FTD for calendar month m was then computed as a trap-day weighted average: the sum of flies assigned to month m divided by the total trap-days occurring in that month,

$$\mathrm{FTD}_\mathrm{m}=\sum \mathrm{C}_\mathrm{i,m}/\sum\mathrm{(T}_\mathrm{i}\times \mathrm{D}_\mathrm{i,m}),$$

where D_i_,ₘ is the count of days from inspection interval i that fall within month m. If an inspection interval crossed a month boundary, captures were apportioned between months in proportion to days spent in each month (equivalently assuming a constant daily capture rate within the interval). Monthly uncertainty was reported as the standard error of the inspection-interval FTD values that contributed trap-days to that month.

Note: summing daily FTD across a month produces a cumulative monthly catch per trap (flies trap⁻¹ month⁻¹); here we report monthly mean FTD (flies trap⁻¹ day⁻¹) to facilitate comparisons across months and sites.

### Gap-filling of missing months with Random Forests

Because trapping was interrupted during several periods, a prediction-based gap-filling procedure using Random Forest was applied to produce continuous monthly FTD time series [26–29]. At SUA, missing months occurred between November 2007 and March 2008. At Nyandira, gaps occurred between late 2006 and late 2008. Among the transect sites, short gaps of one to two months were present at Kibundi, Langali, and Visada. A complete site-by-month dataset was constructed for the respective study periods of each group of sites (October 2005–February 2012 for SUA and Nyandira; October 2008–February 2012 for the four transect sites), retaining observed monthly FTD values and flagging unsampled months as missing.

For each site and month, climatic covariates were included using observed data were available (TMA and data-logger data for SUA; data-logger data for Nyandira and transect sites) and supplementary gridded or reanalysis climate data for periods with missing observations. Temporal variables were included to capture seasonal and inter-annual structure. Predicted FTD values were generated only for the explicitly identified gap periods and merged with observed values to produce a single continuous monthly series for each site. All imputed months were clearly flagged and distinguished from observed data in subsequent analyses.

### Data analysis

All analyses were conducted on monthly Fly-per-Trap-per-Day (FTD) records of *Z. cucurbitae* from six localities, linked with monthly temperature, rainfall, relative humidity, and site elevation. Data processing and statistical analyses were performed in Python (≥ 3.10) using standard scientific libraries, including pandas and NumPy for data handling [30], scikit-learn for machine-learning imputation [31], statsmodels for regression modelling [32], and Matplotlib for visualization [33]. Monthly time series were assembled on a complete locality × month grid so that non-sampling months were explicitly represented and treated consistently across sites.

Missing FTD values were imputed only for visualization and selected time-series analyses using a Random Forest model trained on observed months [34], incorporating locality, calendar time, trap effort, climate covariates, and elevation as predictors. Model performance was evaluated using a held-out test split. Imputed values were used only to support visualization and continuity in explicitly labelled analyses and were not treated as independent observations for hypothesis testing. Temporal trends were assessed with the non-parametric Mann–Kendall trend test and Sen’s slope [35], applying a common trend window (August 2008–February 2012) for sites with shorter records. Seasonal and inter-annual variability in abundance was examined using log(1 + FTD) and non-parametric comparisons across months and years. Differences among sites were tested using the Kruskal–Wallis test followed by Dunn’s post-hoc comparisons with Holm adjustment.

Associations between FTD and climatic variables were explored using Pearson correlations within focal sites and across all localities. Predictability and persistence were characterized through peak/trough timing metrics, lag-1 correlations of annual means, and Spearman trends across years. Temperature trends were estimated using seasonality-controlled regression with month fixed effects to derive annual warming rates for SUA and Nyandira. To assess whether population levels were stable or volatile between years, lag-1 correlation of annual mean abundance were computed.

## Results

### Long term seasonal dynamics of *Z. cucurbitae*

**Fig. 2:**
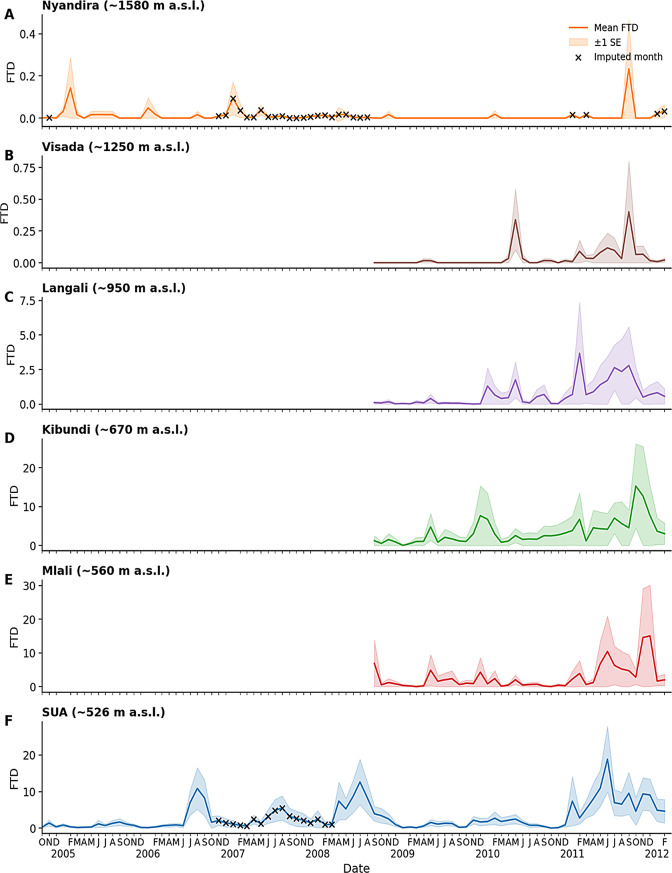
**A–F**: Seasonal abundance dynamics of *Z. cucurbitae* at all study sites

*Zeugodacus. cucurbitae* exhibited pronounced spatial heterogeneity in both number of occurrences and persistence, resulting into three abundance regimes that corresponded broadly with altitude, across sites. High-intensity systems (SUA, Kibundi, Mlali), all located at lower to mid-altitudes, were characterised by recurrent seasonal peaks concentrated in mid-year months (May–August), with occasional late-year amplification (Fig. 2A - F). These sites showed strong interannual variability, culminating in a regional surge during 2011 when maximum values reached approximately 18–19 FTD at SUA and 14–15 FTD at Mlali. Peak periods were associated with marked increases in within-site variability in abundance, reflected by greater fluctuation in FTD (Fig. 2A -F).

In contrast, the high-altitude site (Nyandira) maintained consistently low abundance throughout the monitoring period, with only brief and weak seasonal elevations. Similarly low levels were observed at Visada. These sites lacked sustained population build-up and rarely exceeded 0.4 FTD. Intermediate dynamics were observed at Langali, where detectable abundance emerged later in the monitoring period and remained modest, without sustained high-density phases. Across all sites where population build-up occurred, peak timing was broadly consistent around the mid-year seasonal window, whereas trough periods dominated early- and late-year months (Fig. 2A - F).

### Seasonal predictability and interannual persistence of *Z. cucurbitae* abundance

**Fig. 3:**
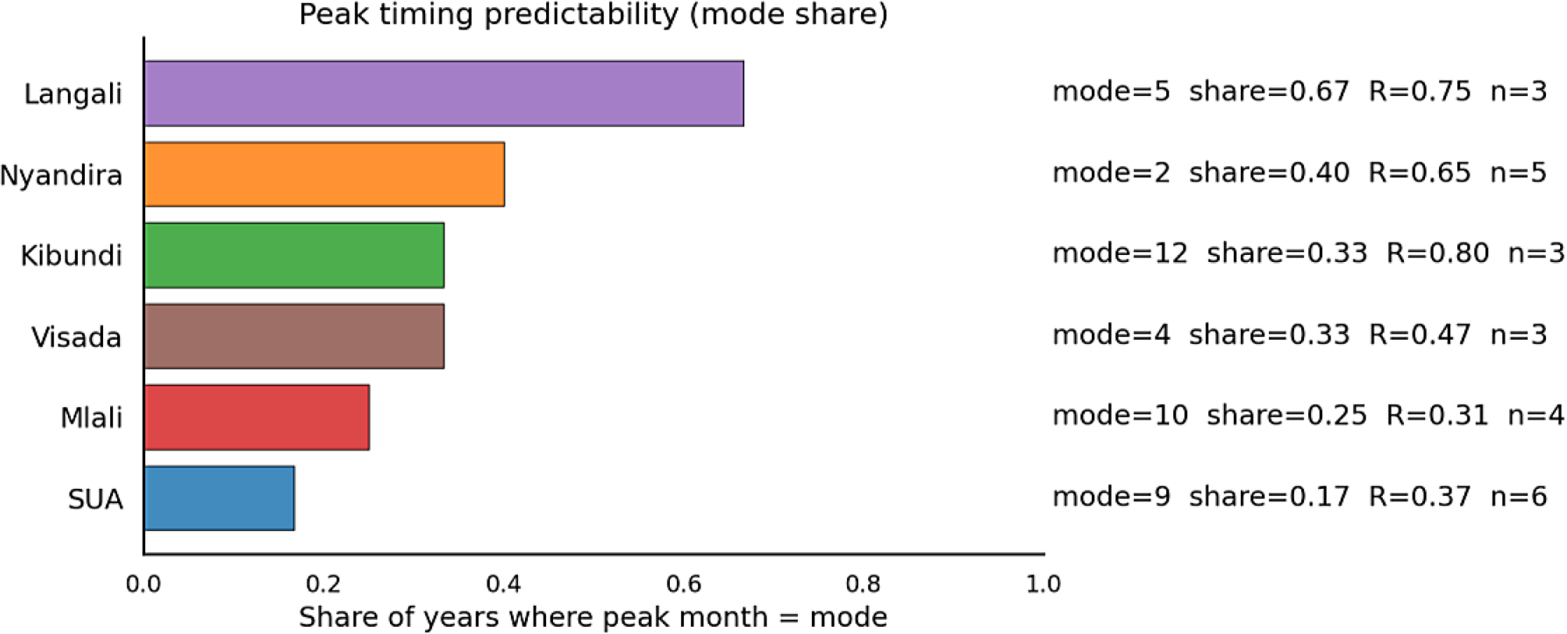
Seasonal predictability of peak abundance across monitoring sites (mode, proportion + Circular concentration index (R))

**Fig. 4:**
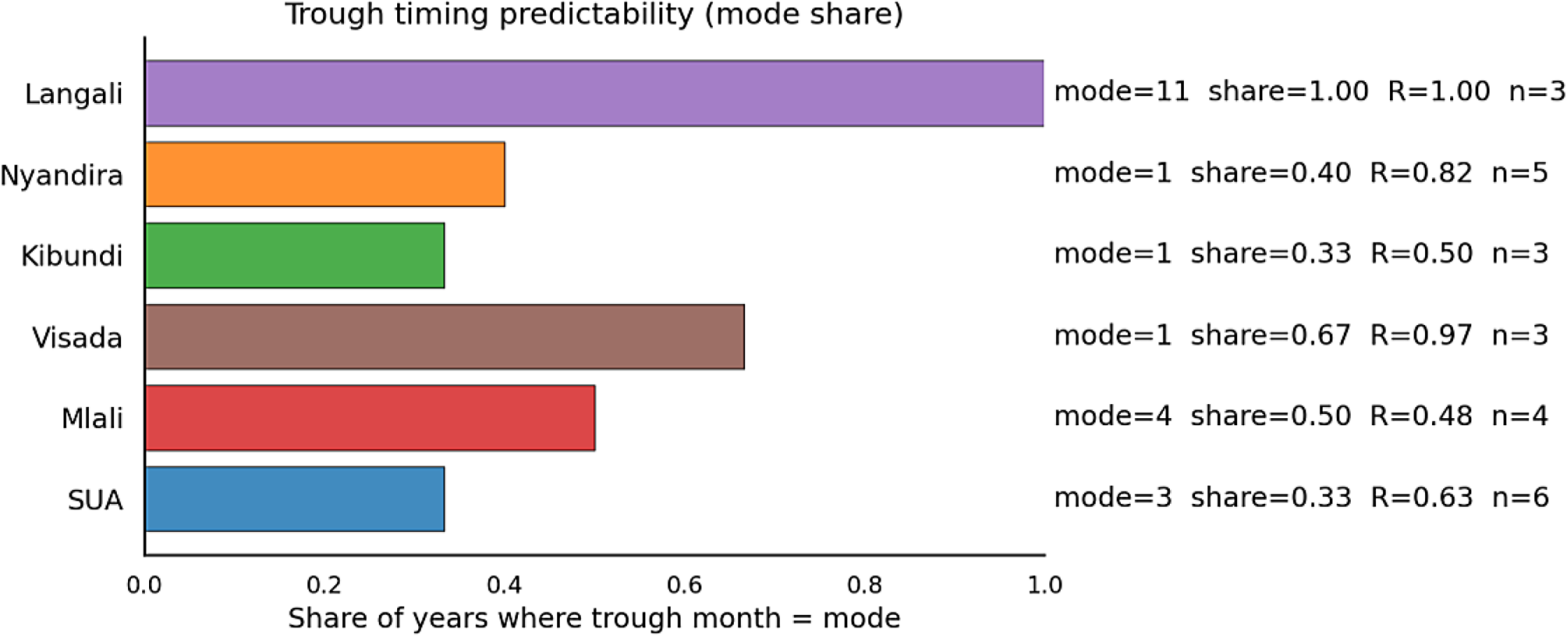
Seasonal consistency of minimum abundance across monitoring sites (mode, proportion + Circular concentration index (R))

Seasonal structure further differentiated sites. Langali exhibited consistent peak timing, reflecting a stable seasonal cycle. In contrast, SUA showed highly variable peak months, consistent with outbreak-driven dynamics. Other sites displayed intermediate regularity (Fig. 3). Trough timing was more consistent than peak timing across sites, indicating stronger seasonal constraint during low-abundance periods. Nyandira and Visada maintained stable suppression phases despite low overall abundance (Fig. 4).

### Long-term directional (monotonic) trends in *Z. cucurbitae* abundance in individual sites

**Fig. 5:**
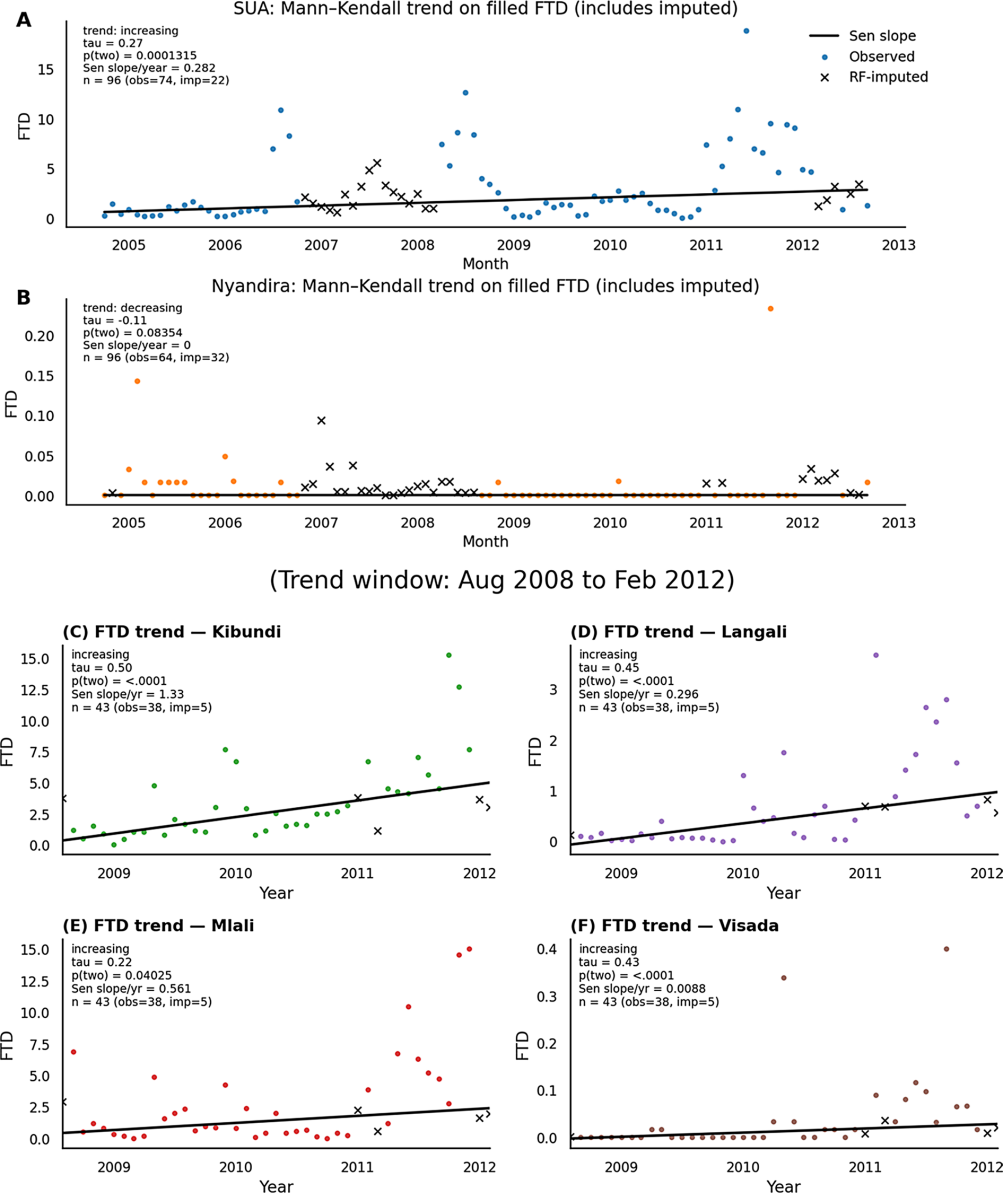
(**A**–**F**): Long-term directional (monotonic) trends in *Z. cucurbitae* abundance across additional monitoring sites (2008–2012) (Mann–Kendall trend test + Sen’s slope estimator)

Long-term trajectories differed among sites and were broadly structured by altitude. At the lower-altitude site (SUA), abundance increased steadily over time. Similar increases were observed at Kibundi, Langali, and Mlali, indicating gradual population expansion at lower and mid-altitude locations (Fig. 5A – F). In contrast, the high-altitude site (Nyandira) remained consistently low and showed no sustained increase, reflecting strong elevation-related constraint. Although Visada showed a statistical upward trend, absolute abundance remained negligible (Figs. 3 and 4). At the annual scale, steady multi-year increases were evident at Kibundi and Langali. However, SUA and Mlali, despite pronounced seasonal peaks—did not exhibit consistent annual growth, demonstrating episodic amplification rather than continuous accumulation.

### Similarity of directional trends among sites

**Fig. 6:**
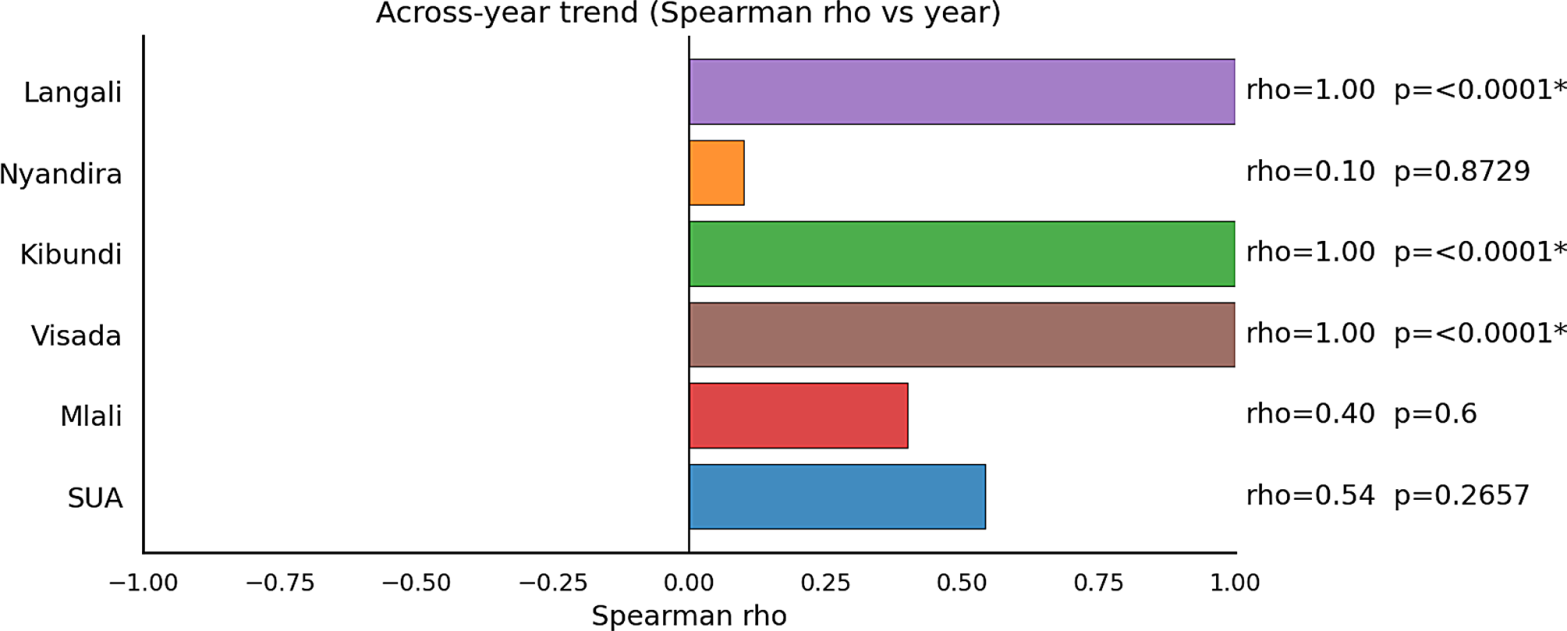
Directional (monotonic) change in annual mean *Z. cucurbitae* abundance (Spearman’s rank correlation test)

To determine whether the increases observed at individual sites reflected similar directional changes over time rather than site-specific dynamics, we examined the similarity of monotonic trends among sites. Spearman’s rank correlations of annual mean abundance across years were used to assess directional consistency. Langali, Kibundi, and Visada exhibited identical strong increasing monotonic trends (ρ = 1.00, *p* < 0.0001 for each), indicating consistent multi-year increases in annual mean FTD despite differences in absolute abundance (Fig. 6). In contrast, Nyandira showed no evidence of a long-term increase (ρ = 0.10, *p* = 0.8729). Similarly, SUA and Mlali displayed non-significant correlations despite pronounced episodic peaks, indicating that their dynamics were driven by irregular surges rather than sustained directional change.

### Intra and inter-annual seasonal dynamics of *Z. cucurbitae*

#### Intra and inter annual patterns

To disentangle short-term seasonal fluctuations from longer-term temporal shifts, we compared abundance variation within years (among months) and across years over the monitoring period.

**Fig. 7:**
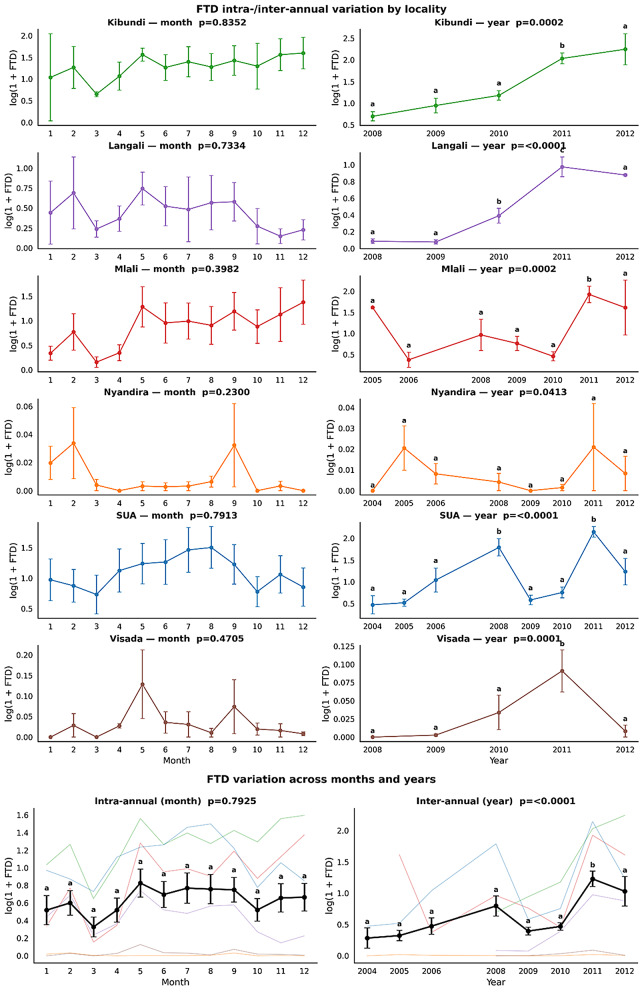
Site-level comparison of seasonal and inter-annual variation in *Z. cucurbitae* abundance (Kruskal–Wallis test + Dunn’s post-hoc test (Holm adjustment))

When abundance was summarised across all sites using a log (1 + x) transformation, differences among months within a year were not statistically significant (*p* = 0.79). In contrast, differences among years were significant (*p* < 0.0001), with higher abundance in 2011 and 2012 compared with earlier years (Fig. 7).

At the locality level, month-of-year effects on abundance were similarly weak at all sites (all *p* ≥ 0.23), whereas year effects were consistently significant, including Kibundi (*p* = 0.0002), Mlali (*p* = 0.0002), Visada (*p* = 0.0001), and SUA (*p* < 0.0001; Fig. 7). Together, these results show that inter-annual variation, rather than within-year seasonality, primarily structured abundance patterns over the monitoring period.

#### Persistence of abundance

Strong positive persistence was observed at Langali, Kibundi, and Visada, each showing lag-1 correlations of 1.00, indicating that years with relatively higher abundance tended to be followed by similarly high years, and low years tended to remain low.

**Fig. 8:**
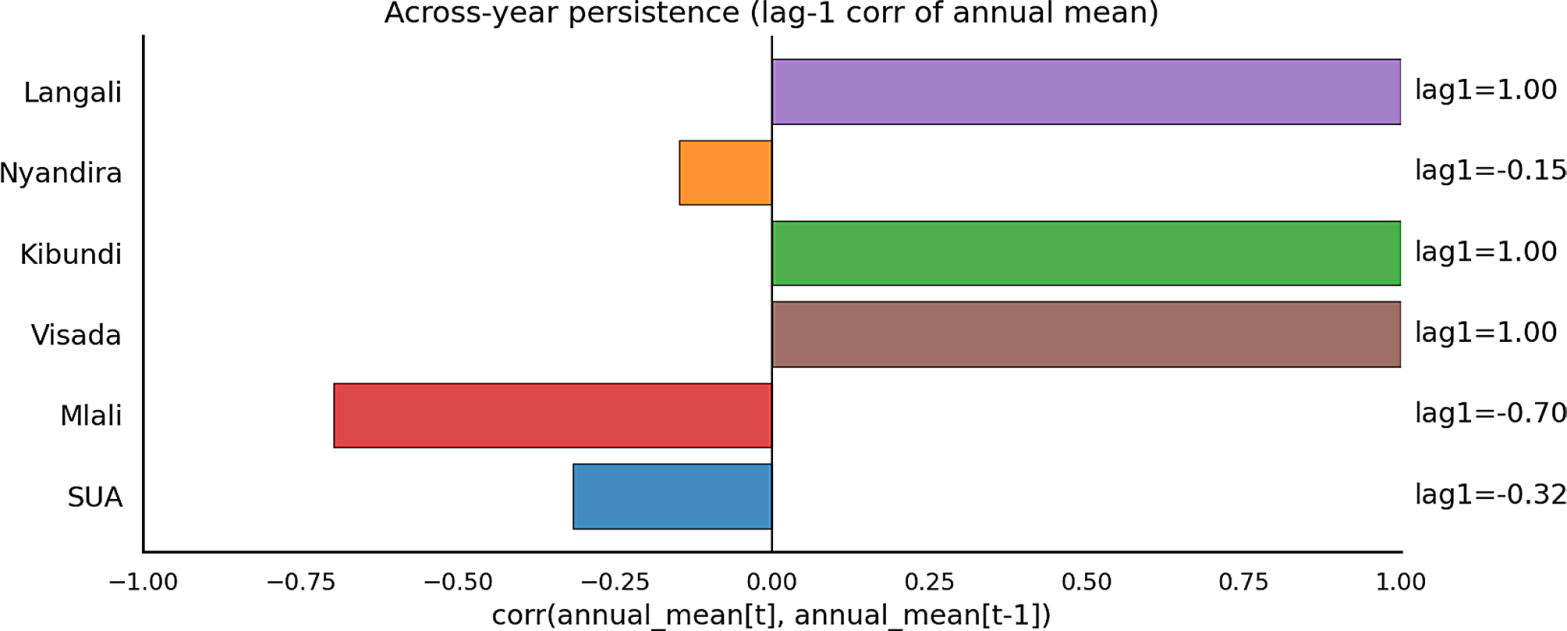
Inter-annual persistence of *Z. cucurbitae* population levels (Pearson lag-1 autocorrelation)

In contrast, Mlali exhibited negative persistence (− 0.70), implying strong year-to-year reversals in abundance and suggesting a more unstable or episodic population trajectory. SUA also showed weak negative persistence (− 0.32), consistent with its irregular outbreak behavior, while Nyandira displayed near-zero persistence (− 0.15), reflecting chronically low abundance with limited inter-annual structure (Fig. 8).

### Altitudinal variations in *Z. cucurbitae* abundance

**Fig. 9:**
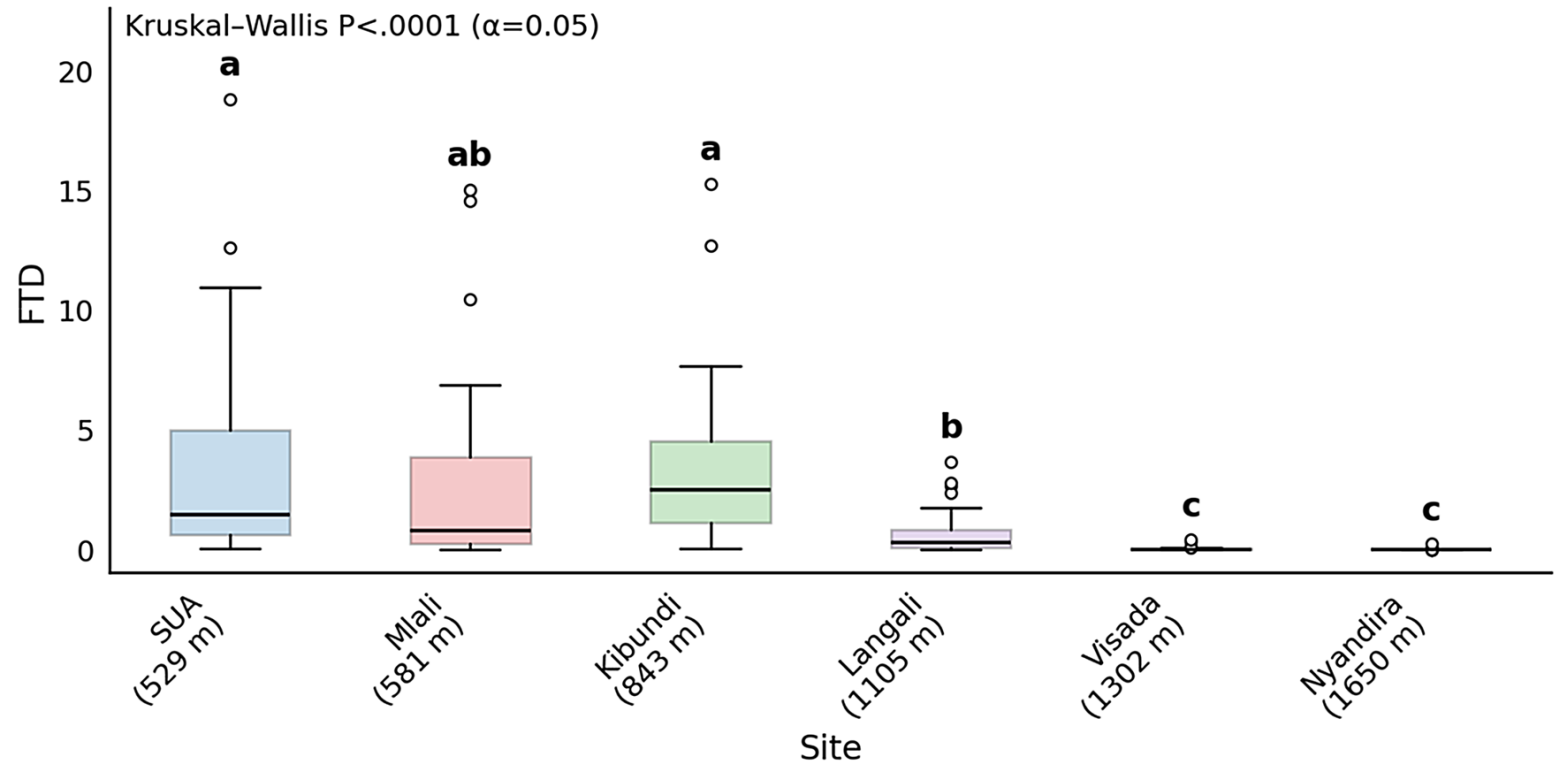
Altitudinal differences in *Z. cucurbitae* abundance across monitoring sites (Kruskal–Wallis test + Dunn’s post-hoc test with Holm adjustment)

**Fig. 10:**
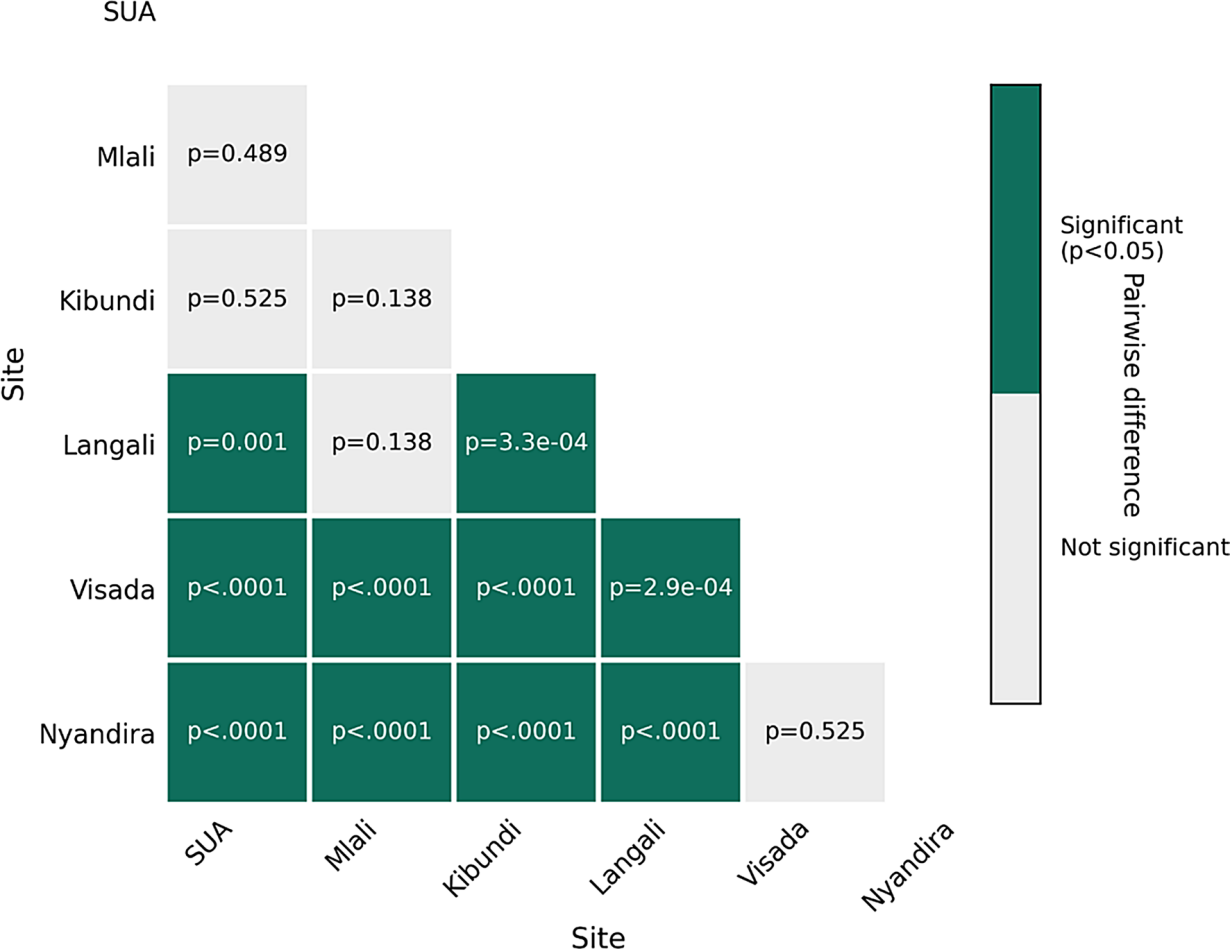
Pairwise comparisons of abundance among altitudinal zones (Dunn post-hoc pairwise differences, Holm-adjusted)

FTD differed strongly among sites (Kruskal–Wallis *p* < 0.0001; Fig. 9). Post-hoc Dunn tests with Holm adjustment confirmed that the two highest-elevation sites (Visada and Nyandira) did not differ from each other (adjusted *p* = 0.525), but each was significantly lower than the three lower-elevation sites SUA, Mlali and Kibundi (all adjusted *p* < 0.0001; Fig. 10). Among the lower-elevation sites, FTD did not differ significantly between SUA and Mlali (*p* = 0.489) or SUA and Kibundi (*p* = 0.525), whereas Langali was lower than SUA (*p* = 0.001) and Kibundi (*p* = 3.3 × 10⁻⁴) (Fig. 10).

### Climate drivers of *Z. cucurbitae* abundance

**Fig. 11:**
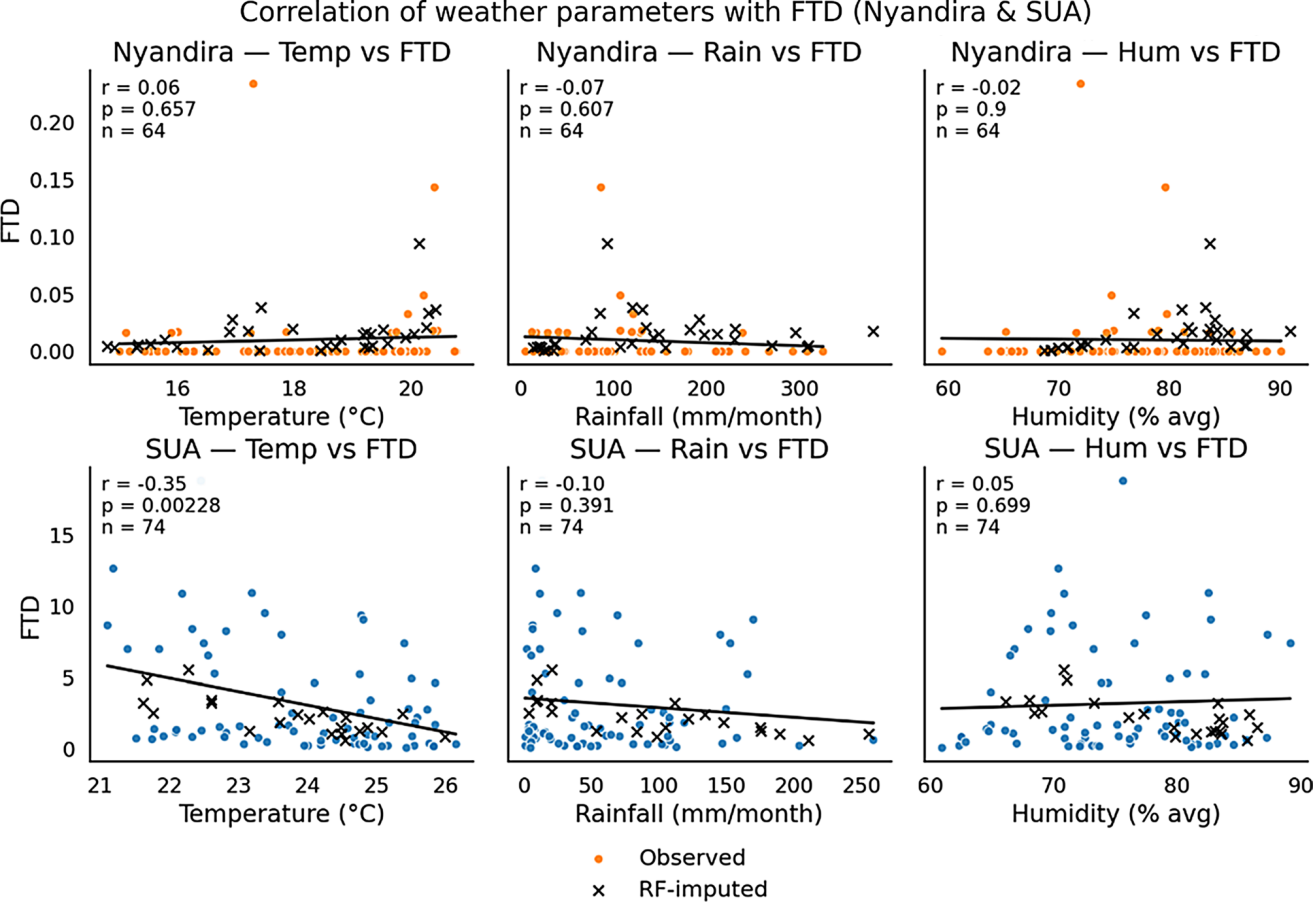
Relationships between climate variables and *Z. cucurbitae* abundance at contrasting altitudes

At Nyandira, pairwise correlations between FTD and contemporaneous climate variables were near zero and not statistically significant (temperature *p* = 0.657; rainfall *p* = 0.607; *p* = 0.90). At SUA, temperature was negatively associated with FTD (*r* = − 0.35; *p* = 0.00228), while rainfall and humidity showed no evidence of association (*p* > 0.5) (Fig. 11).

**Fig. 12:**
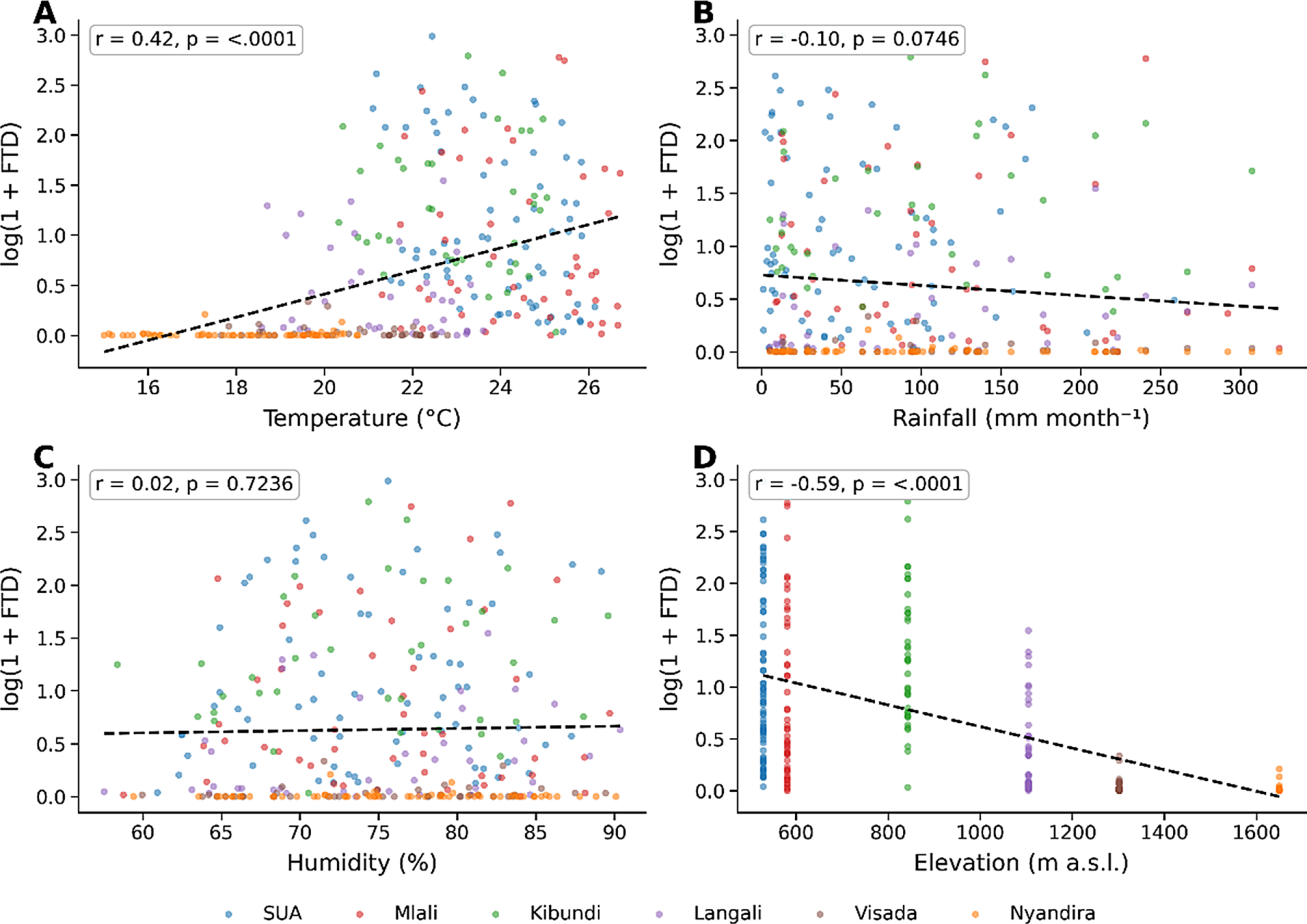
Temperature and elevation gradients in *Z. cucurbitae* abundance across sites

When pooling across all localities, abundance was positively associated with temperature (*r* = 0.42; *p* < 0.0001) and negatively associated with elevation (*r* = − 0.59; *p* < 0.0001), while rainfall (*r* = − 0.10; *p* = 0.0746) and humidity (*r* ≈ 0.02; *p* = 0.7236) remained weak (Fig. 12A-D). The monitored localities spanned a pronounced lowland–highland gradient, which provided a useful geographic framework for comparing site-level differences in abundance.

### Seasonality-controlled temperature trends

**Fig. 13:**
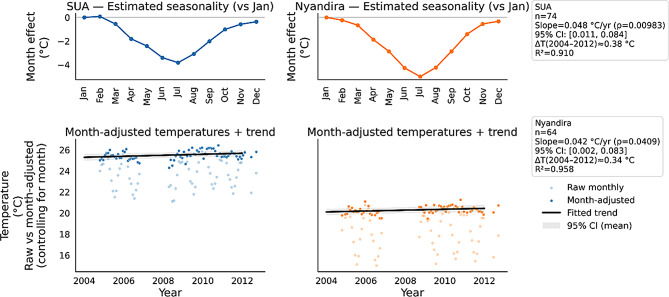
Long-term warming trends at low- and high-altitude monitoring sites (Ordinary Least Squares (OLS) regression with month fixed effects)

After controlling for month-of-year (fixed effects), temperature increased significantly at both SUA and Nyandira. The estimated trend at SUA was 0.048 °C yr⁻¹ (*p* = 0.00983; 95% CI [0.011, 0.084]; R² = 0.910), corresponding to an approximate warming of 0.38 °C from 2004 to 2012. Nyandira showed a similar warming rate of 0.042 °C yr⁻¹ (*p* = 0.0409; 95% CI [0.002, 0.083]; R² = 0.958), corresponding to ~ 0.34 °C, over the same period (Fig. 13).

## Discussion

Our study demonstrated that the population dynamics of *Z. cucurbitae* were structured primarily by inter-annual variation, with relative increases often observed, while month-of-year effects were weak, whereas year-to-year differences dominated abundance patterns. This indicates that temporal dynamics were driven more by processes operating at annual scale than by regular seasonal recurrence.

Marked spatial heterogeneity further shaped these dynamics as abundance declined along the lowland–highland gradient, with lower values at higher elevations and greater variability at lower elevations. Peak timing was weakly predictable at some locations and variable across sites, whereas low-abundance periods were more consistent. Differences in persistence and trends revealed contrasting regimes, from stable trajectories to episodic peaks. Together, these patterns indicate that dynamics arise from inter-annual forcing interacting with site-specific constraints rather than uniform seasonal cycles.

Across Africa, *Z. cucurbitae* is strongly associated with warm, low-altitude agro-ecosystems and is rare or absent at higher elevations [12, 13, 25]. Geurts et al. [25] showed declining abundance with altitude, while Mwatawala et al. [12] reported absence of the species at a high-altitude site of Nyandira (~ 1580 m a.s.l.). These findings confirm that temperature and elevation are primary constraints, although recent evidence complicates this general picture. Tarimo et al. [17] reported relatively high infestation levels of *Z. cucurbitae* at mid-altitude sites in the same region when trapping occurred within cucurbit fields, suggesting that host availability can partly offset temperature constraints. Although the elevation of range in Tarimo et al. [17] did not extend to the higher altitudes examined in the present study, their findings nonetheless highlight the importance of local host availability in shaping realised population densities.

Despite this general pattern, seasonal peaks vary among regions reflecting differences in climatic context and host availability. In Benin, Gnanvossou et al. [13] observed population increases from August and peaks between October and January, with positive associations with humidity and negative associations with minimum temperature. In contrast, Mwatawala et al. [12] found peak populations during the long dry season (June–October), and reported negative relationships between temperature and humidity, with no rainfall effect. These results show high densities can occur under contrasting climatic regimes. Results from Zida et al. [36] show strong land-use effects, with higher densities in cultivated areas and frequent absence in natural habitats; interactions among site, biotope, and time highlight the role of agricultural systems in outbreaks.

These contrasts are best explained by host availability interacting with rainfall, while temperature sets broad physiological limits. Mwatawala et al. [12] suggested that rainfall-driven host availability is more important than temperature. In Tanzania, cucurbit production often increases after the rainy season, leading to population peaks in the dry period. In Benin, host plants and suitable fruits are more abundant during humid months, resulting in wet-season population peaks [13]. Tarimo et al. [17], working in the same study area, demonstrated that fruit fly infestation in cucurbit fields was closely tied to crop phenology and the timing of growing seasons, further supporting the central role of host availability in driving local abundance patterns.

An important limitation of our study is that trapping was not conducted in designated cucurbit fields, so catches likely reflect dispersing adults rather than field populations. Trap counts may be influenced by host proximity, availability, and dispersal distances, and may therefore differ from actual field populations. Future studies should integrate trapping with direct host sampling and phenology data [17].

Beyond these methodological constraints, environmental factors remain central to explaining observed patterns, with temperature remaining a fundamental constraint, with experimental studies showing optimal development within specific thermal ranges [8, 9], while extreme heat imposes stress and cooler high-altitude environments restrict persistence [12, 25]. These effects may be further shaped by geographic population structure, as genetic differentiation and limited gene flow [2, 6] suggesting that regional variation in climatic sensitivity. In addition, species interactions may influence seasonal dynamics, with niche partitioning reported [37, 38]. However, other *Dacus* species were not a focus of this study and interspecific effects could not be assessed.

These findings have direct implications for integrated pest management indicating that control strategies should be climate-responsive and locally adapted, and interventions should align with peak activity periods [24]. In regions with dry-season peaks, management should be intensified immediately after the rainy season, whereas in regions with wet-season peaks, interventions should begin at the onset of rains.

The strong association of *Z. cucurbitae* with cultivated habitats [36] indicates that management should focus on agricultural landscapes as evidence show that continuous or irrigated production can sustain populations year-round [4]. Crop sanitation and reduction of off-season host availability therefore remain critical components of effective management.

Seasonal variation in natural enemy activity [14] further suggests that biological control efficacy will vary and should be incorporated into IPM planning. Overall, the seasonal dynamics of *Z. cucurbitae* arise from interacting effects of climate, altitude, host availability, land use, and species interactions, supporting climate-responsive, site-specific pest management.

## Conclusion

The sustained high abundance at low- and mid-altitude sites (SUA, Kibundi, Mlali), the regional amplification observed in 2011–2012, and the steady directional increases at Kibundi and Langali indicate that management efforts should prioritise these climatically permissive zones, particularly during mid-year build-up periods (May–August). Future studies should integrate host availability, land-use change, and finer-scale climate variables to clarify the drivers of episodic outbreaks and to model potential upslope range shifts under continued warming. Complementing para-pheromone-based surveillance with direct host fruit sampling, as conducted by Tarimo et al. [17], will be essential for linking landscape-level trap catches to actual infestation dynamics. Such work will strengthen early warning systems and support spatially targeted, climate-responsive management of *Z. cucurbitae*.

## Electronic Supplementary Material

Below is the link to the electronic supplementary material.

Supplementary Material 1

Supplementary Material 2

## Data Availability

The datasets used and analysed during the current study are available from the corresponding author on reasonable request.
