# Supplementary material for: Long-term monitoring reveals climate-driven seasonal dynamics of Zeugodacus cucurbitae (Coquillett) in Tanzania and prospects for pest control
Source: BMC Ecol Evol. 2026 Jun 1;26:67. doi: 10.1186/s12862-026-02536-6 (PMC13449795; doi:10.1186/s12862-026-02536-6)
Supplement: Supplementary file 1 [file 12862_2026_2536_MOESM1_ESM.docx]

## Supplementary Figures

**Figure S1: Monthly temperature (^o^C) trends across sites**

**Figure S2: Monthly rainfall (mm/month) trends across sites**

**Figure S3: Monthly relative humidity (%) trends across sites**

**Figure S4: Seasonality of temperature (^o^C) across sites (monthly climatology)**

**Figure S5: Seasonality of rainfall (mm/month) across sites (monthly climatology)**

**Figure S6: Seasonality of relative humidity (%) across sites (monthly climatology)**
