# Supplementary material for: Long-term monitoring reveals climate-driven seasonal dynamics of Zeugodacus cucurbitae (Coquillett) in Tanzania and prospects for pest control
Source: BMC Ecol Evol. 2026 Jun 1;26:67. doi: 10.1186/s12862-026-02536-6 (PMC13449795; doi:10.1186/s12862-026-02536-6)
Supplement: Supplementary file 2 [file 12862_2026_2536_MOESM2_ESM.docx]

# Supplementary Tables

**Table S1: Description of the study area (Morogoro region)**

| **Zone** | **Characteristics** |
| --- | --- |
| Mountain zone | Altitude: > 600 meters asl; Average rainfall: 800mm – 2500mm p.a.; Average temperature: 18^o^c |
| Plateau | Altitude: 300-600 meters asl; Average rainfall: 700mm – 1200mm p.a.; Average temperature: 25^o^c |
| River valleys and basins | Altitude: < 300 meters asl; Average rainfall: 900mm – 1400mm p.a.; Average temperature: 30^o^c |

**Table S2: Crops grown in the study area**

| Location | Fruits grown |
| --- | --- |
| SUA horticulture unit  (6°51′11″S, 37°39′27″E) | Mango, *Mangifera indica* L., Tangerine *Citrus reticulata* Blanco, Lemon *Citrus limon* (L.) Burman f., Sweet orange, *Citrus sinensis* (L.) Osbeck., Breadfruit, *Artocarpus altilis* (Parkins.) Fosb., Avocado, *Persea americana* Miller., Governors’ plum, *Flacourtia indica* (Burman f.) Merr., Guava, *Psidium guajava* L., Grape fruit, *Citrus paradisi* Macfad., Soursop, *Annona muricata* L., Cherimoya, *Annona cherimola* Miller, Pomelo, *Citrus grandis* (Burman) Merr., Star fruit, *Averrhoa carambola* L., Grapes, *Vitis vinifera* L., Passion fruit, *Passiflora edulis f.s. flavicarpa*, Loquat, *Eriobotrya japonica* (Thunb.) Lindley, Kumquat, *Fortunella margarita* (Thunb.) Swingle, Jackfruit, *Artocarpus heterophyllus* Lam., Pawpaw, *Carica papaya* L., and Jew plum, *Spondias cytherea* Sonn occur |
| Nyandira  (7°05′03″S, 37°34′37″E) | Apple, *Malus* spp*.,* Peach, *Prunus persica* (L.) Batsch., Plum *Prunus salicina*  Lindley, Stawberry, *Fragaria* spp., Coffee, *Coffea canephora* Pierre ex A. Froehner, Feijoa *Feijoa sellowiana* (O. Berg) O. Berg., Guava, *Psidium* *guajava* L:, Nectarines, *Prunus persica* (L.) Batsch, Loquat, Cherimoya, and Avocado. |
